# Recent trends and therapeutic potential of phytoceutical‐based nanoparticle delivery systems in mitigating non‐small cell lung cancer

**DOI:** 10.1002/1878-0261.13764

**Published:** 2024-11-26

**Authors:** Adam Haysom‐McDowell, Keshav Raj Paudel, Stewart Yeung, Sofia Kokkinis, Tammam El Sherkawi, Dinesh Kumar Chellappan, Jon Adams, Kamal Dua, Gabriele De Rubis

**Affiliations:** ^1^ Discipline of Pharmacy, Graduate School of Health University of Technology Sydney Ultimo Australia; ^2^ Australian Research Consortium in Complementary and Integrative Medicine, School of Public Health University of Technology Sydney Ultimo Australia; ^3^ Centre for Inflammation Centenary Institute, Faculty of Science, School of Life Sciences University of Technology Sydney Australia; ^4^ Department of Life Sciences, School of Pharmacy International Medical University Kuala Lumpur Malaysia

**Keywords:** cancer pathways, drug delivery system, nanomedicine, non‐small cell lung cancer, phytoceutical

## Abstract

Lung cancer is the leading cause of cancer death globally, with non‐small cell lung cancer accounting for the majority (85%) of cases. Standard treatments including chemotherapy and radiotherapy present multiple adverse effects. Medicinal plants, used for centuries, are traditionally processed by methods such as boiling and oral ingestion, However, water solubility, absorption, and hepatic metabolism reduce phytoceutical bioavailability. More recently, isolated molecular compounds from these plants can be extracted with these phytoceuticals administered either individually or as an adjunct with standard therapy. Phytoceuticals have been shown to alleviate symptoms, may reduce dosage of chemotherapy and, in some cases, enhance pharmaceutical mechanisms. Research has identified many phytoceuticals' actions on cancer‐associated pathways, such as oncogenesis, the tumour microenvironment, tumour cell proliferation, metastasis, and apoptosis. The development of novel nanoparticle delivery systems such as solid lipid nanoparticles, liquid crystalline nanoparticles, and liposomes has enhanced the bioavailability and targeted delivery of pharmaceuticals and phytoceuticals. This review explores the biological pathways associated with non‐small cell lung cancer, a diverse range of phytoceuticals, the cancer pathways they act upon, and the pros and cons of several nanoparticle delivery systems.

## Graphical abstract

Bioactive molecules from plants, known as phytoceuticals, have been shown to upregulate anticancer pathways and downregulate pro‐cancer pathways associated with non‐small cell lung cancer. However, low solubility, poor absorption, and hepatic metabolism result in poor biovavailability. Utilising advanced nanoparticle delivery systems to encapsulate phytoceutical molecules to improve biovailability has the potential to improve lung cancer treatment.

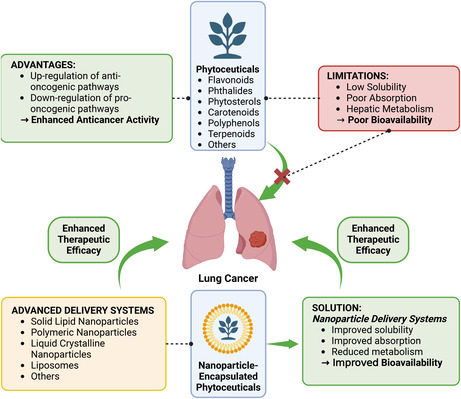

**Abbreviations**
ADAR1: RNA‐specific adenosine deaminase
AMPK: adenosine monophosphate‐activated protein kinase
ATF4: activating transcriptional factor 4
Bax: Bcl‐2 associated protein X
Bcl‐2: B‐cell leukaemia‐lymphoma 2 protein
Bcl‐3: B‐cell leukaemia 3
Bcl‐xL: B‐cell lymphoma extra long
Bcl‐xS: B‐cell lymphoma extra short
CD4: tumour‐infiltrating cluster of differentiation 4
CD8: tumour‐infiltrating cluster of differentiation 8
CDKI 1A/1B: cyclin‐dependent kinase inhibitors 1A/1B
CDKN2D: cyclin dependent kinase inhibitor 2D
CHOP: CCAAT‐enhancer‐binding protein homologous protein
c‐Myc: cellular Myc
COX‐2: cyclooxygenase‐2
e1F2a: eukaryotic initiation factor 2α
EGFR: epidermal growth factor receptor
EMT: epithelial‐mesenchymal transition
FAK‐ERK 1/2: focal adhesion kinase‐extracellular signal‐regulated kinase 1/2
GLOBOCAN: Global Cancer Observatory
H2AX: H2A histone family member X
HBA: hydroxybenzoic acids
iCAM‐1: intercellular adhesion molecule 1
IFN‐γ: interferon‐gamma
IL‐1β: interleukin‐1‐beta
IL‐2: interleukin‐2
IL‐3: interleukin‐3
IL‐6: interleukin‐6
IRE1: inositol‐requiring enzyme 1 alpha
JAK2: janus kinase
Kras‐ERK: Kirsten rat sarcoma virus‐extracellular signal‐regulated kinase
KRT‐18: keratin 18
LC: lung cancer
LCN: liquid crystalline nanoparticle
LPS: lipopolysaccharide
LUAD: lung adenocarcinoma
MMP‐2: matrix metalloproteinase 2
MMP‐9: matrix metalloproteinase 9
mTOR: mechanistic target of rapamycin
MTT: molecular targeted therapy
NF‐kB: nuclear factor kappa‐light‐chain‐enhancer of activated B cells
NSCLC: non‐small cell lung cancer
p38 MAPK: mammalian p38 mitogen‐activated protein kinase
p53: tumour protein p53
p‐Akt: phosphorylated Akt
PARP1: poly(ADP‐ribose) polymerase 1
pCA: *p*‐coumaric acid
PD‐L1: programmed death‐ligand 1
p‐ERK: phosphorylated extracellular signal‐regulated kinase
PERK: protein kinase R (PKR)‐like endoplasmic reticulum kinase
PI3K/Akt: phosphatidylinositol 3‐kinase and serine and threonine kinase
PIP3: phosphoinositide‐3 kinase (PI3K)‐regulated pathway
PMN: polymeric nanoparticle
PRISMA: Preferred Reporting Items for Systematic Reviews and Meta‐Analyses
PTEN: phosphatase and tensin homologue deleted on chromosome 10
PTX: paclitaxel
PUMA: p53 upregulated modulator of apoptosis
ROS: reactive oxygen species
SCLC: small cell lung cancer
Sirt1: sirtuin
SLN: solid lipid nanoparticle
sPLA2‐Iia: group II secretory phospholipase A2
STAT3: signal transducer and activator of transcription
TAM: tumour associated macrophage
TC: tumour cell
TCM: Traditional Chinese Medicine
TME: tumour microenvironment
TNF‐α: tumour necrosis factor‐α
TRIB3: tribbles pseudokinase 3
VEGF‐C: vascular endothelial growth factor C

## 1 Background

**Fig. 1:**
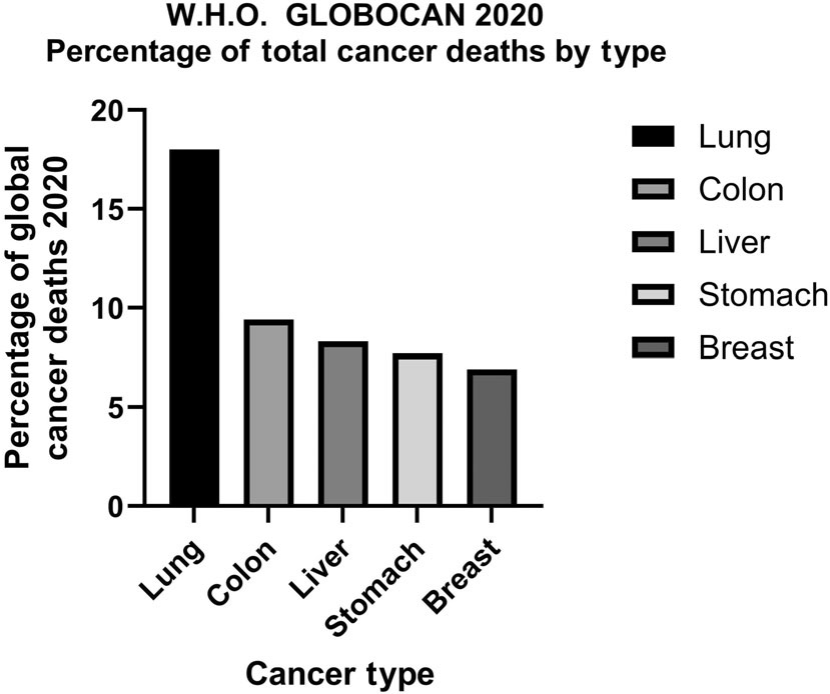
Global deaths by cancer type as a percentage. Data extrapolated from “Global Cancer Statistics 2020: GLOBOCAN Estimates of Incidence and Mortality Worldwide for 36 Cancers in 185 Countries.” Figure created in graphpad prism v10.2.3 (GraphPad Software, Boston, MA, USA).

According to the World Health Organisation Global Cancer Observatory (GLOBOCAN) Registry 2020, the leading five causes of cancer deaths worldwide are lung (18%), colon (9.4%), liver (8.3%), stomach (7.7%), and female breast (6.9%) [1] (Fig. 1). The total number of lung cancer deaths from over 2.2 million diagnoses, resulted in ~1.8 million deaths from the disease in 2020 [1].

It is not surprising, then, that in Australia, lung cancer is also the leading cause of cancer death, with more than 14 500 new cases in 2022 [2]. With a 5‐year survival rate between 10% and 20%, non‐small cell lung cancer (NSCLC) accounts for 85% of all cases [1, 3]. The remaining cases are categorised as small cell lung cancer (SCLC) [4]. NSCLC, may be further classified as squamous cell carcinoma, large cell carcinoma, or lung adenocarcinoma (LUAD) [5]. Smoking, including tobacco and e‐cigarettes, is the major risk factor for NSCLC, with increasing age and family history also playing a significant role in the development of the disease [6]. Air pollution is also a concerning risk factor worldwide, with the 2023 World Air Quality Report indicating that only 10 out of 134 countries meet the World Health Organisation's guidelines [6, 7]. Nevertheless, exposure to risk factors does not guarantee oncogenesis, nor can the absence of risk factors preclude it, with 80% of cancers being of unknown aetiology [8].

The literature pertaining to the use of nanoparticle delivery systems for cancer is increasing. However, more research is warranted. To compare the available literature pertaining to research investigating nanoparticle systems with the aforementioned top five cancer types, the authors completed a database search (April 2024) of four common medical repositories. The databases searched were PubMed, Cumulative Index to Nursing and Allied Health Literature (CINAHL), Exerpta Medical Database (EMBASE), and Scopus. The search terms “nanoparticle delivery AND *cancer type*” were used, where *cancer type* indicates the name of the cancer in question (e.g. “nanoparticle delivery AND lung cancer”). Considering NSCLC is the most common form of lung cancer, a subset search using “non‐small cell lung cancer” for the cancer type was also examined. In all databases, a date range of 2014 onwards was selected. The limitations of this simplified database search are recognised in that the Preferred Reporting Items for Systematic Reviews and Meta‐Analyses (PRISMA) would be desirable [9]. This presents an opportunity for future reviews to perform robust analyses of the literature.

**Fig. 2:**
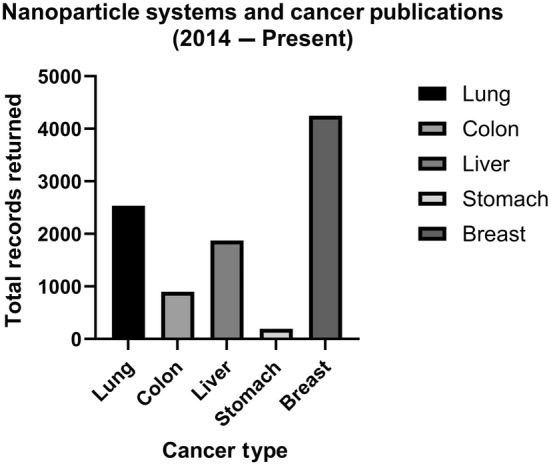
Publications of nanoparticle systems and cancer. Records were searched within PubMed, CINAHL, EMBASE, and Scopus databases (April 2024) using the search terms “nanoparticle delivery AND cancer type”, where “cancer type” indicates the name of the cancer in question (e.g. “nanoparticle delivery AND lung cancer”) from 2014 onwards. The cancer types are noted as the five leading causes of cancer death globally by GLOBOCAN. Figure created in graphpad prism v10.2.3.

The results illustrated in Fig. 2 imply that the majority of cancer‐related nanoparticle delivery system research is for breast cancer, followed by lung, liver, colon, with stomach cancer returning the fewest records. NSCLC records (not shown) amounted to 24% of all lung cancer articles. It is suggested that while lung cancer has the highest mortality, it is falling behind in the area of nanoparticle delivery research, highlighting the need for further research investigating nanoparticle delivery systems for lung cancer, and specifically NSCLC.

The information within this review may be utilised for further research to develop revolutionary systems of anticancer phytoceuticals delivery for the treatment of NSCLC.

### 1.1 Current treatment interventions and related challenges

Treatment options for NSCLC include surgery with neoadjuvant or adjuvant therapy, radiation therapy, chemotherapy, immunotherapy, molecular targeted therapy (MTT), as well as different combinations of these types of interventions [10, 11]. Despite the numerous therapeutic options available, ~70% of diagnoses are with advanced disease stages IIIB or IV, with 5‐year survival rates of 10% and 1%, respectively [12]. Additionally, NSCLC is known to rapidly acquire chemotherapy resistance [13]. MTT is an attractive choice of treatment and has several advantages compared to other interventions, such as targeting pathways and mechanisms that promote tumour cell (TC) apoptosis, ameliorate angiogenesis within the tumour microenvironment (TME), and suppress TC proliferation and migration [14]. However, no intervention is without risk of physical, psychological, and financial adverse effects. Surgery may result in post‐surgical pain, with chemotherapy and radiation patients experiencing loss of appetite, skin irritation, shortness of breath, dysphagia, fatigue, nausea, vomiting, hair loss, oesophagitis, and pleural effusion [15, 16].

Psychological distress presenting as depression and anxiety is common in lung cancer patients [17, 18]. Cross‐sectional studies have identified depression in at least 58% of lung cancer patients, as well as a positive association of increasing anxiety with higher numbers of chemotherapy sessions [19]. Furthermore, the economic burden of NSCLC on society is significant. While current treatment‐related cost data are not available from Australia, in the US alone, individual costs range from US$3700 to US$5800 per month [3, 20]. Elsewhere, it has been shown that treatment cost is directly associated with disease stage [3].

### 1.2 Major limitations in cancer treatment: therapeutic resistance

Lung cancer patients often face therapeutic resistance, resulting in extended treatment times, cancer recurrence, metastasis, and a reduced survival rate [21]. Chemoresistance may arise from multiple factors including, but not limited to, drug efflux and influx changes, epigenetic changes, DNA damage, epithelial‐mesenchymal transition (EMT) changes, and crosstalk of TME components [21, 22].

Therapeutic resistance is not limited to chemotherapy. Radiation therapy may result in poorer clinical outcomes due to radiation‐induced DNA damage [21], modification of cell cycle progression genes, and the dysregulation of signalling pathways such as the phosphatidylinositol 3‐kinase and serine and threonine kinase (PI3K/Akt) and the Janus kinase/signal transducer and activator of transcription/B‐cell leukaemia‐lymphoma 2 protein/B‐cell lymphoma extralarge (JAK2/STAT3/Bcl‐2/Bcl‐XL) survival pathways [23].

While MTT may have fewer side effects compared to conventional chemotherapy [24], it does not come without its own challenges. MTT may be ineffective due to one or more points of failure. The molecular target must be precise, and inappropriate identification of disease aetiology, pathogenesis, pathways, or biomarkers pose the risk of poor outcomes [25]. Additionally, despite initial accurate molecular targeting, treatment failure occurs if TCs develop alternate pathways of propagation that do not coincide with the original biomarkers targeted by the MTT [14]. Therefore, novel interventions that prevent disease progression are not only integral to the survival rate, but also key to reducing unpleasant side effects, psychological suffering, and economic burden.

### 1.3 Growing relevance of phytoceutical‐based treatment options

Plant‐based medicines, such as those employed in Traditional Chinese Medicine (TCM), have been used to treat tumours for centuries. The earliest known record of “malignant sores” and “swelling without ulceration” was documented by practitioners in the Qin Dynasty (221–207 B.C.E.) [26]. More recently, isolated bioactive molecules from plants (phytoceuticals) such as artemisinin, berberine, boswellic acid, 18‐β glychrrhetinic acid, emodin, fisetin, oridonin, hydroxysafflor yellow A, ligustilide, ginsenoside Rg3, and taraxasterol have been shown to provide promise in the treatment of cancer [27, 28, 29, 30, 31, 32, 33, 34, 35]. Nevertheless, maximising the bioavailability of phytoceutical compounds is a key challenge. Factors such as limited bioavailability due to poor water solubility, low permeability, reduced absorption across the intestinal wall, stability in the acidic gastric environment, and first‐pass metabolic effects all limit bioavailability [36], and hence the clinical efficacy of phytoceuticals. To overcome these limitations of phytoceuticals, nanodelivery methods such as polymeric nanoparticles, solid lipid nanostructured carriers, liquid crystalline nanoparticles, liposomes, and microemulsions have been thoroughly investigated to improve absorption, reduce side effects, and lower the required dosage [37].

Considering the physical, emotional, and financial impact of NSCLC on patients, the present review explores the growing relevance of phytoceuticals in this field. In particular, the review identifies the mechanisms of a diverse range of phytoceuticals in targeting the various pathways associated with NSCLC oncogenesis and proliferation, with an exploration of various nanoparticle systems as a potentially revolutionary means to improve the bioavailability, and therefore the potential for clinical translation of phytoceuticals.

## 2 Non‐small cell lung cancer pathways and genes

Lung cancer is responsible for the majority of cancer‐related deaths, with NSCLC accounting for the vast majority (85%) [38]. Of the NSCLC subtypes, LUAD is the predominant type, accounting for 40% of tumours [38, 39]. Depending on the histology, LUAD is further classified into lepidic, acinar, papillary, micropapillary, or solid [40]. With such a variety of NSCLC phenotypes, molecular biology and histopathology assessment is vital for correct pathological diagnosis and implementation of appropriate treatment strategies [5]. Once the tumour physiology and characteristics have been distinguished, specific molecular and genetic pathways can be targeted to inhibit development and metastasis [14].

### 2.1 Cancer development and pro‐oncogenic pathways

Cancer initiation, or oncogenesis and can be the result of cell signalling dysregulation, gene mutations, oxidative stress, inflammation, and defective immune system mechanisms [41, 42, 43]. A variety of pro‐oncogenic pathways, which support cancer growth, lead to other aspects of tumour development, including various hallmarks of cancer such as cell proliferation, angiogenesis, maturity of the TME, inhibition of apoptosis, and metastasis [42, 43, 44].

In lung cancer, the most frequent oncogene mutation occurs on the epidermal growth factor receptor (EGFR) gene, which results in overexpression in up to 80% of NSCLC cases, with Ki‐ras2 Kirsten rat sarcoma viral oncogene homologue (Kras) being the most common oncogene‐driven subtype of LUAD [38, 42]. Cyclooxygenase‐2 (COX‐2), which regulates the production of inflammatory‐mediating prostaglandins, is also overexpressed in LUAD and has been the subject of a number of preclinical trials [45]. COX‐2 appears to be a worthy target due to its multifaceted functions in multiple pathways, including oncogenesis, cell proliferation, metastasis, and chemoresistance [45, 46].

Mutations of the PI3K‐Akt pathway are present in 3–5% of all cancers and lead to uncontrolled cell proliferation [47]. Similarly, abnormal accumulation of phosphatase and tensin homologue deleted on chromosome 10 (PTEN) (an Akt trigger), cellular Myc (c‐Myc), mammalian p38 mitogen‐activated protein kinase (p38 MAPK), and nuclear factor kappa‐light‐chain‐enhancer of activated B cells (NF‐kB) result in aberrant cell proliferation [33, 48, 49, 50]. Once a tumour accumulates, ~1 million cells, it requires additional nutrients and oxygen, which it achieves by promoting angiogenesis and through the development of the TME [51, 52]. The pathways contributing to these processes include Bcl‐xL, COX‐2, matrix metalloproteinase‐2 (MMP‐2), and matrix metalloproteinase‐9 (MMP‐9) [45, 53, 54, 55].

During the processes of proliferation, angiogenesis, and establishment of the TME, tumours require strategies to mitigate the immune system from triggering apoptosis, and this is where the Bcl‐2, focal adhesion kinase‐extracellular signal‐regulated kinase1/2 (FAK‐ERK1/2), NF‐kB, and programmed death‐ligand 1 (PD‐L1) pathways work to maintain tumour cell viability [34, 41, 50, 56]. If the immune system succeeds in reducing cancer cell viability by triggering apoptosis, or perhaps restricting nutrients by inhibiting angiogenesis, the tumour may metastasise [57]. Here, RNA‐specific adenosine deaminase (ADAR1) and intercellular adhesion molecule 1 (iCAM‐1) join forces with COX‐2 and EGFR/Kras as cell migration enablers [58, 59].

### 2.2 Anti‐oncogenic pathways

While the inventory of pro‐oncogenic pathways is extensive, there is an equally broad list of antioncogenic pathways that work to inhibit tumour angiogenesis, constrain cell proliferation, and promote apoptosis [60, 61, 62, 63]. Triggering cancer cell apoptosis is a function of multiple pathways, including adenosine monophosphate‐activated protein kinase (AMPK), Bcl‐2 associated protein X (Bax), Caspase‐3, ‐7, ‐8, ‐9, H2A histone family member X (H2AX), and protein kinase R (PKR)‐like endoplasmic reticulum kinase (PERK)/Eukaryotic Initiation Factor 2α (eIF2α)/activating transcriptional factor 4 (ATF4)/CCAAT‐enhancer‐binding protein homologous protein (CHOP) [60, 61, 63, 64, 65].

Numerous factors have multifaceted antioncogenic pathways. AMPK regulates mechanistic target of rapamycin (mTOR) signalling and inhibits cancer proliferation by downregulating COX‐2 while simultaneously inducing p53 expression [60], as does p53 upregulated modulator of apoptosis (PUMA) [66]. In turn, p53 upregulates Bax, which triggers apoptosis and suppresses cell proliferation [35, 64]. There are also pathways that hold dual roles, both positive and negative for cancer, often depending on the specific molecular and cellular context. The protein E‐cadherin has been studied for its function in suppressing tumour growth signalling [67]. When expression of E‐cadherin and cyclin‐dependent kinase inhibitors 1A/1B (CDKI 1A/1B) is enhanced, there is an arrest of the cell cycle and a suppression of proliferation in lung cancer [32, 68]. However, while metastatic cancers are often found to coincide with a reduced expression of E‐cadherin, its overexpression has been implicated in an aggressive form of breast cancer [62, 67].

Tumour necrosis factor‐α (TNF‐α) is a known proinflammatory cytokine associated with multiple cancer hallmarks including cancer cell proliferation, angiogenesis, invasion, and metastasis, notably as a prominent NF‐kB activator [69, 70]. Conversely, TNF‐α is able to bind to tumour necrosis factor receptor 1 (TNF receptor 1), a death domain receptor with the ability to induce apoptosis, making it a potential anticancer target [71]. A number of natural and synthetic compounds, including 17‐allylamino‐17‐demethoxygeldanamycin, parthenolide, wogonin, and luteolin have been shown to inhibit TNF‐α activated NF‐kB pathways, resulting in TNF‐α induced cytotoxicity of tumour cells [71, 72, 73, 74].

**Fig. 3:**
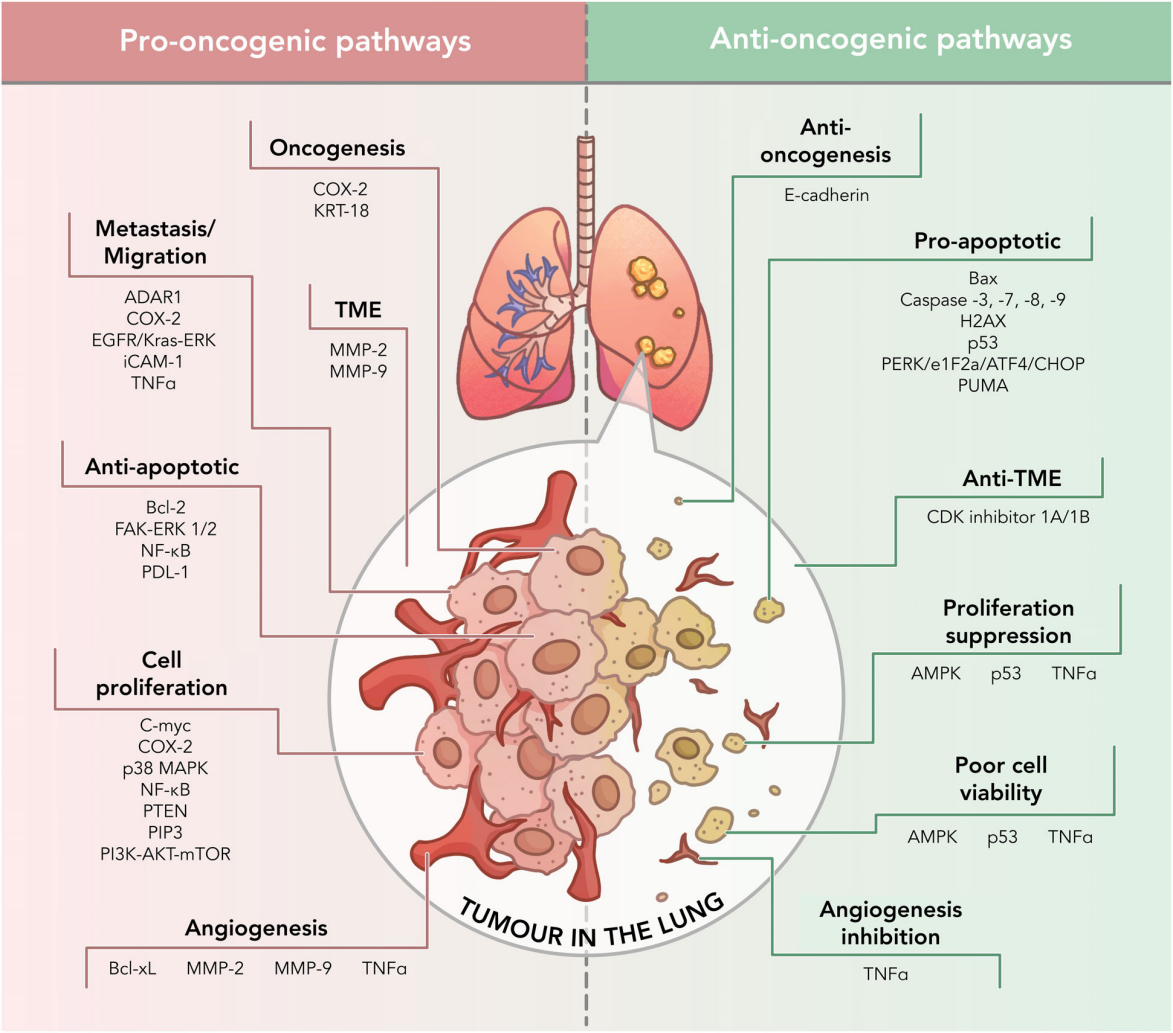
Pro‐oncogenic and anti‐oncogenic pathways associated with the hallmarks of lung cancer (including NSCLC) such as angiogenesis, cell proliferation, and metastasis. Proteins and genes shown are activating transcriptional factor 4 (ATF4), adenosine monophosphate‐activated protein kinase (AMPK), B‐cell leukaemia‐lymphoma 2 protein (Bcl‐2), B‐cell lymphoma extralong (Bcl‐xL), Bcl‐2 associated protein X (Bax), caspase‐3, ‐7, ‐8, ‐9, CCAAT‐enhancer‐binding protein homologous protein (CHOP), cellular Myc (C‐myc), cyclin‐dependent kinase inhibitors 1A/1B (CDK inhibitor 1A/1B), cyclooxygenase‐2 (COX‐2), e‐cadherin, epidermal growth factor receptor (EGFR), eukaryotic initiation factor 2α (e1F2a), focal adhesion kinase‐extracellular signal‐regulated kinase 1/2 (FAK‐ERK 1/2), H2A histone family member X (H2AX), intercellular adhesion molecule 1 (iCAM‐1), keratin 18 (KRT‐18), kirsten rat sarcoma virus‐extracellular signal‐regulated kinase (Kras‐ERK), mammalian p38 mitogen‐activated protein kinase (p38 MAPK), matrix metalloproteinase 2 (MMP‐2), matrix metalloproteinase 9 (MMP‐9), mechanistic target of rapamycin (mTOR), nuclear factor kappa‐light‐chain‐enhancer of activated B cells (NF‐kB), p53 upregulated modulator of apoptosis (PUMA), phosphatase and tensin homologue deleted on chromosome 10 (PTEN), phosphatidylinositol 3‐kinase and serine and threonine kinase (PI3K‐AKT), phosphoinositide‐3 kinase (PI3K)‐regulated pathway (PIP3), programmed death‐ligand 1 (PDL‐1), protein kinase R (PKR)‐like endoplasmic reticulum kinase (PERK), RNA‐specific adenosine deaminase (ADAR1), tumour necrosis factor‐α (TNF‐α), and tumour protein p53 (p53). Figure created with procreate v5.3.9 (Savage Interactive, Hobart, Australia) and adobe illustrator v2023 (Adobe Inc., San Jose, CA, USA).

Figure 3 illustrates the various pro‐oncogenic and antioncogenic pathways associated with the hallmarks of lung cancer (including NSCLC) such as angiogenesis, cell proliferation, and metastasis.

## 3 Anticancer molecular mechanisms of phytoceuticals against NSCLC

Historically, herbal medicines utilised whole plants or complete parts thereof, such as flowers, seeds, leaves, bark, or roots [75]. In 1805, the first isolated compound from a medicinal plant was extracted. This was the phenanthrene alkaloid from opium, called morphine [76, 77]. Following this, a diverse range of phytochemical classes and molecular compounds, many with anticancer properties, has been identified [78, 79, 80]. These phytochemical groups include polyphenols, terpenes, sulphurous compounds, nitrogenous compounds, phthalides, phytosterols, and carotenoids, each with characteristic molecular structures [80, 81, 82, 83, 84]. According to their molecular structure, bioavailability is determined by properties such as lipophilicity, molecule size, electron distribution, hydrogen bonding, and structural flexibility [85]. In the next section, we explore various phytochemical groups and describe their molecular structures.

### 3.1 Phytochemical groups

The polyphenols group, identified by having at least one hydroxyl group attached to an aromatic ring, encompasses no less than 8000 compounds, each grouped into subclasses based on the number of phenol rings and binding structural elements [86]. Within the polyphenols group, phenolic acids are compounds with one carboxylic acid group [86, 87]. Flavonoids are identified as having two aromatic rings bonded by three carbon atoms, whereas stilbenes are identified with two phenyl moieties linked by a two‐carbon methylene bridge [86]. Lignans are polyphenolic binaphthalene compounds, more specifically, stereospecific dimers of monolignols [88, 89].

**Fig. 4:**
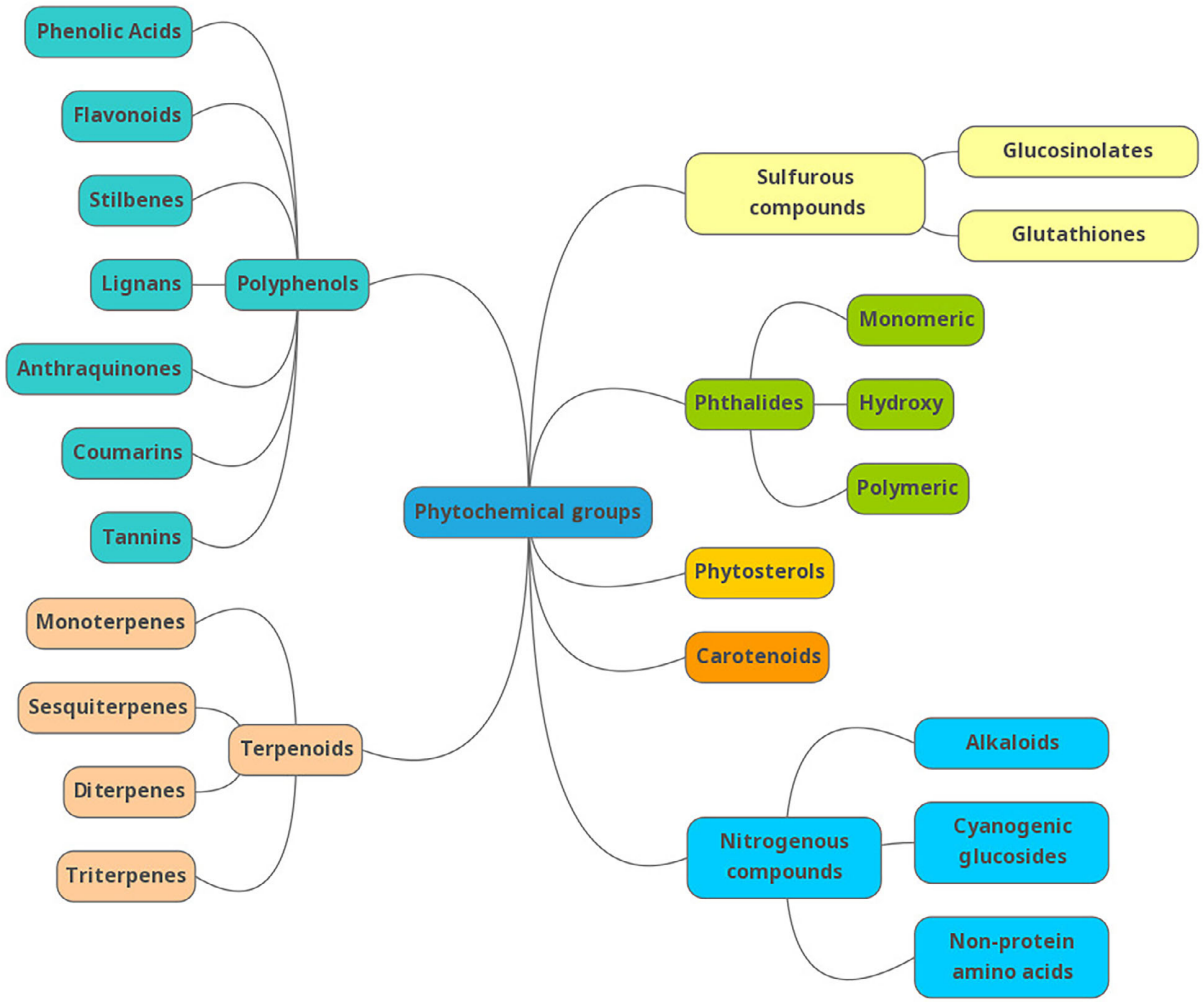
Phytochemical groups and their associated subcategories from which phytoceutical compounds with anticancer mechanisms may be extracted. Figure created with mindmup v4.44.65 (Sauf Pompiers Limited, Leigh‐On‐Sea, UK).

Terpenoids are a separate phytochemical group to polyphenols and are modified versions of terpenes, with five carbon isoprene units and varying functional groups [82]. Nitrogenous and sulphurous compounds, as the names suggest, contain at least one nitrogen or sulphur atom, respectively, in their molecular structure [90, 91]. The phthalides group contains at least 133 compounds, which are further categorised into monomeric, hydroxy, and polymeric subgroups, with the majority having a monomeric 1(3H)‐isobenzofuranone core structure [80]. Additional groups include phytosterols, carotenoids, coumarins, and tannins [81, 83, 84]. Figure 4 illustrates and summarises these aforementioned phytochemical groups.

### 3.2 Polyphenols

Polyphenols are cited as offering valuable protection against a variety of diseases such as diabetes, cardiovascular diseases, neurodegenerative diseases, and cancer [86]. Polyphenols undergo molecular modification during absorption due to conjugation and hepatic metabolism, hence the resulting serum concentration is not equitable to the intake from food [86]. Phenolic acids, mostly contained within the pericarp of fruits, vegetable leaves, and seeds, are divided into cinnamic acids and hydroxybenzoic acids (HBA), with gallic acid (GA) being the most researched of these [87]. Within the cinnamic acid subgroup is ferulic acid (FA), caffeic acid, *p*‐coumaric acid (*p*CA), and sinapic acid [86]. Studies show that GA and *p*CA, from *Dimocarpus longan*, have high levels of intestinal absorption and bioavailability [86, 87]. While *p*CA, has a higher bioavailability profile than GA [92, 93], the latter appears to be of interest, as it exhibits dual functions of enhancing antioncogenic pathways while inhibiting pro‐oncogenic factors in NSCLC cell lines [94].

Within the flavonoids group, quercetin, myricetin, kaempferol, hydroxysafflor yellow A (HSYA), genistein, daidzein, and fisetin are commonly studied [32, 81, 86, 95]. HSYA, a quinochalcone‐C‐glycoside from *Carthamus tinctorious flos*, has been researched for post‐stroke cognitive impairment and has been shown to downregulate pro‐oncogenic factors [31, 95, 96].

Fisetin, found in apples and grapes amongst other fruits, has its highest concentrations in *Fragaria × ananassa*, otherwise known as strawberries [32]. Fisetin has been investigated for its function in modulating cancer‐preventative pathways including inflammation, apoptosis, and angiogenesis [32, 97, 98]. Furthermore, fisetin has shown potential as an adjunct to chemotherapy. When applied with paclitaxel (PTX) or cyclophosphamide, the result is an increased apoptosis of the NSCLC adenocarcinoma cell line (A549) cells and inhibited angiogenesis within the TME, respectively [97, 98].

Emodin is an anthraquinone extracted from *Radix et rhizome rhei* (rhubarb root), which also offers synergistic actions when applied with PTX [99]. Emodin together with PTX has been shown to enhance expression of pro‐apoptotic factors, while decreasing pro‐oncogenic factors in A549 cells [99].

While resveratrol is the most investigated stilbene, its structural analogue, pterostilbene, has a four times higher bioavailability [86, 100]. Sourced from *Vaccinium sect cyanococcus* (blueberries), pterostilbene's enhanced bioavailability is due to the molecule containing two methoxy groups, resulting in enhanced lipophilic absorption [100]. Pterostilbene has been shown to upregulate antioncogenic pathways in a variety of lung cancer cell lines [101, 102].

The lignans, honokiol and magnolol, are separated only by a change to the hydroxyl group position, resulting in two beneficial compounds is [103]. Isolated from the bark of *Magnoliae officinalis*, they can both cross the blood–brain‐barrier to exhibit neuroprotective effects [103, 104, 105]. Both have been shown to downregulate pro‐oncogenic factors, resulting in reduced cell viability in several NSCLC cell lines [106, 107].

### 3.3 Terpenes

Terpenes, often found in grapes, flowers, fruits, wood, and roots, have a five‐carbon isoprene unit 2‐methyl‐1,3‐butadiene [108]. Terpenoids, which are modified terpenes with different functional groups, are further categorised as monoterpenes, sesquiterpenes, diterpenes, sesterpenes, triterpenes, and meroterpenes [82]. All have exhibited anticancer mechanisms [27, 34, 82, 109, 110].

Diterpenes are found in species such as *Rabdosia rubescens*, *Gingko biloba*, *Coffea arabica*, as well as *Propolis*, a biproduct produced by honeybees [30, 111, 112, 113]. The tetracyclic diterpene, oridonin, is known for its antiinflammatory, cardioprotective, hepatoprotective, and neuroprotective mechanisms [114]. In chronic lung diseases, mechanisms associated with inflammation lead to airway remodelling, characterised by excessive extracellular matrix (ECM) deposition, EMT, and airway obstruction [115]. Over time, the production of lung fibroblasts and ECM deposition lead to stiffening and loss of elasticity known as fibrosis [116], which is known to increase the risk of oncogenesis [117]. Oridonin's benefits to NSCLC stem not only from ameliorating pulmonary fibrosis, but its ability to inhibit the EMT process and promote cancer cell apoptosis [30, 114, 118]. The sesquiterpene artemisinin, from *Artemisia annua*, has traditionally been used as an antimalarial [119]. However, more recently, artemisinin has been shown to inhibit metastasis of Lewis Lung Cancer (LLC) and promote apoptosis of A549 cells [27, 109].

Within the triterpenes group, approximately 30 ginsenosides have been isolated from *Panax ginseng* [120]. While ginsenoside Rb1 (Rb1) and ginsenoside Rg1 (Rg1) are the most abundant saponins in *Panax ginseng* [120], ginsenoside Rg3 (Rg3) has been shown to inhibit cisplatin resistance‐associated PD‐L1 expression in A549 cells [34].

### 3.4 Nitrogenous compounds

This group is divided into alkaloids, cyanogenic glucosides, and non‐protein amino acids [81]. Alkaloids, further classified into isoquinoline, indole, and pyridine‐like groups, are known to have poor solubility and low bioavailability [121, 122]. Berberine, an isoquinoline sourced from the rhizome of *Coptis chinensis* and other *Berberis* species, has long been used in traditional medicines for a variety of conditions, including inflammatory bowel disease, type 2 diabetes, bacillary dysentery, pertussis, and eczema [122, 123, 124].

There has been a marked increase in global interest of identifying the interactions of berberine and various cancers over the last two decades. This is illustrated by Yuan *et al*. [125], who show that published articles relating to berberine and tumours rose from <10 in the 12 months of 2002 to almost 180 in 2018, with NSCLC being the 4th highest researched.

### 3.5 Phthalides

Phthalides are predominantly found in essential oils from species such as *Angelica sinensis*, *Ligusticum chuanxiong*, and *Cnidium officinale* [80, 126]. The monomeric phthalide, ligustilide, is the main phytochemical compound of *A. sinensis* and *L. chuanxiong* [33, 127]. These plants have been used in traditional medicine for menstrual disorders, recovery from cerebral infarction, and wound healing due to their ability to promote angiogenesis, inhibit cell apoptosis, and promote cell growth [128, 129, 130]. While these properties seem counterintuitive to cancer therapy, these cell‐preserving mechanisms of ligustilide do not maintain NSCLC cells in the same manner. Jiang *et al*. [33] demonstrated *in vitro* that ligustilide reduced cell viability and induced apoptosis of A549 and H1299 cells by dampening NSCLC aerobic glycolysis. Conversely, ligustilide did not induce apoptosis of BEAS‐2B cells. This result suggests that ligustilide may be used to inhibit NSCLC cells while preserving healthy bronchial epithelial tissue [33].

Cancer cells benefit from the hypoxic environment of the TME, with tumour hypoxia correlating with advanced malignancy [131, 132]. NSCLC cells take advantage of this environment with energy pathway adaptations for glycolytic metabolism [33]. Ligustilide inhibits NSCLC glycolysis by mediating the PTEN/AKT signalling pathway and increasing caspase‐3, ‐7, resulting in reduced TME angiogenesis and increased tumour cell apoptosis [33]. Ligustilide has a reduced effect on cell viability and proliferation of human lung epithelial cells, suggesting that ligustilide has potential as a targeted therapy for NSCLC [33].

### 3.6 Isolated compounds versus whole extracts

Prior to the isolation of molecular compounds, traditional remedies relied on extracting medicinal components by boiling, decocting as teas, macerating and chewing, and infusing as ointments [133]. It has been argued that methods such as these, using whole plant components, provide a synergy of compounds resulting in multitarget effects [134]. Conversely, it is recognised that some compounds may antagonise each other, thereby inhibiting each other's actions [134]. While examples of each effect are published, it appears that further investigation into whole‐plant synergism and antagonism compared to isolated compounds is warranted [134].

### 3.7 Phytoceutical compounds with actions against NSCLC in the literature

While clinical trials investigating the actions of various phytoceuticals against cancer are limited, a few studies have shown an inverse relationship between higher intake of certain compounds and incidences of cancer [135]. Those studies pertaining to lung cancer and the intake of flavonoids such as epicatechin, catechin, quercetin, and naringin identified that a higher intake resulted in an inverse relationship with the development of the disease [136, 137, 138]. Studies assessing kaempferol have returned mixed outcomes, with Cui *et al*. [138] indicating that intake is associated with fewer lung cancer cases in smokers but not nonsmokers. The meta‐analysis compiled by Tang *et al*. included data from eight prospective studies and four case–control studies encompassing 5073 lung cancer cases and 237 981 noncases. These authors concluded that the highest flavonoid intake was positively associated with a reduced risk of lung cancer. Furthermore, a 10% decrease in the risk of lung cancer was associated with an increased flavonoid intake of 20 mg·day^−1^ [137].

**Fig. 5:**
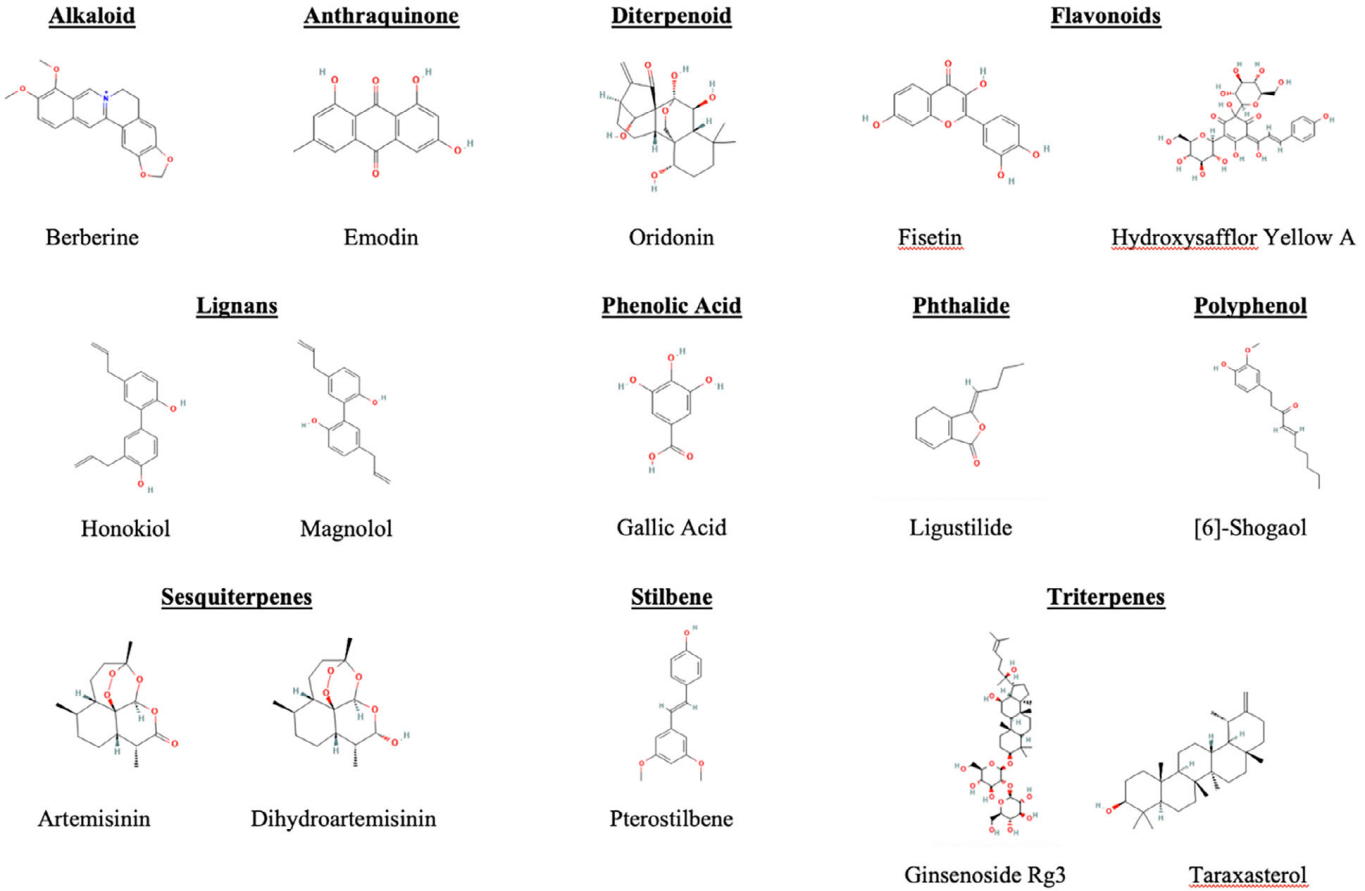
Phytochemical molecular structures of berberine, emodin, oridonin, fisetin, hydroxysafflor yellow A, honokiol, magnolol, gallic acid, ligustilide, [6]‐shogaol, artemisinin, dihydroartemisinin, pterostilbene, ginsenoside Rg3, and taraxastero,l which have mechanisms to downregulate pro‐oncogenic and/or upregulate anti‐oncogenic factors, as expanded in Table 1.

Table 1 outlines an examination of the literature pertaining to numerous isolated compounds and whole plant extracts (WPE), with the phytoceutical molecular structures illustrated in Fig. 5. It is shown that both WPE and isolated compounds can inhibit pro‐oncogenic factors, activate antioncogenic factors, and enhance chemotherapy drug actions. The NSCLC pathways and targets addressed by these WPEs and isolated compounds include many of the hallmarks of cancer, including EMT, tumour proliferation and growth, tumour angiogenesis, tumour metastasis, and cell apoptosis. Elsewhere, isolated compounds have been shown to inhibit cancer‐related pain [139]; however, they have not been included here and may be the subject of future investigations.

**Table 1:** List of studies on phytoceuticals with anticancer (NSCLC) mechanisms to downregulate pro‐oncogenic and/or upregulate anti‐oncogenic factors with the associated pathways/genes

Source	Whole plant extract/isolated compound	Pharmacology/mechanisms	Downregulate (*DR*) pro‐oncogenic factors/upregulate (*UR*) anti‐oncogenic factors	Pathways/gene	Reference
*Angelica sinensis radix*	Ligustilide (isolated compound)	Ligustilide inhibits PTEN/AKT signalling; increases caspase‐3 and ‐7 activity in A549 and human non‐small cell lung carcinoma cell line H1299 (H1299) cells → increased apoptosis and reduced cell viability	*DR* pro‐oncogenic factors and *UR* anti‐oncogenic factors	PTEN/AKT Caspase‐3, ‐7	[33]
	Whole plant extract	Acetone extract → suppresses expression of Bcl‐2 oncoprotein → triggers apoptosis → inhibits A549 cell proliferation	*DR* pro‐oncogenic factors and *UR* anti‐oncogenic factors	Caspase‐3, ‐9 Bcl‐2	[187]
*Artemisia annua*	Whole plant extract	Artemisia annua extract inhibits *in vitro* NSCLC A549 cell viability	Not investigated	Cell viability	[188]
	Dihydroartemisinin (semisynthesised compound)	Dihydroartemisinin encapsulated in hyaluronan nanoparticles → inhibits proliferation and triggers apoptosis of A549 LC cells *in vitro*	Not investigated	Apoptosis	[109]
	Artemisinin (isolated compound)	Artemisinin → inhibits Vascular endothelial growth factor C (VEGF‐C) expression within Lewis LC tumours → inhibits metastasis	*DR* pro‐oncogenic factors	VEGF‐C	[27]
	Artemisinin (isolated compound)	Artemisinin → decreases Interleukin 1‐beta (IL‐1β) induced activation of p38 and VEGF‐C expression → inhibits tumour lymphangiogenesis & metastasis in Lewis Lung Carcinoma	*DR* pro‐oncogenic factors	p38 MAPK VEGF‐C	[27]
*Carthamus tinctorius flos*	Hydroxysafflor yellow A (isolated compound)	Hydroxysafflor yellow A → inhibits iCAM‐1, TNF‐α, IL‐1β and Interleukin‐6 (IL‐6) in Lipopolysaccharide (LPS) induced damaged A549 cells	*DR* pro‐oncogenic factors	P38 MAPK	[31]
	Hydroxysafflor yellow A (isolated compound)	Hydroxysafflor yellow A → attenuates NF‐kB p65 protein nuclear translocation in LPS‐induced damaged A549 cells	*DR* pro‐oncogenic factors	NF‐kB	[31]
	Safflower polysaccharides	Safflower polysaccharide → inhibits PI3K/Akt pathway → induces NSCLC cell apoptosis	*DR* pro‐oncogenic factors	PI3K/Akt	[4]
*Coptis chinensis rhizoma*	Berberine (isolated compound)	Berberine activates AMPK‐p53 → blocks PI3k‐AKT, MAPK or Sirt1 cell growth signalling pathway → inhibits tumour growth	*UR* anti‐oncogenic factors	AMPK p53 PI3k‐AKT MAPK	[189]
	Berberine (isolated compound)	Berberine‐phytantriol liquid crystalline nanoparticles → inhibits A549 cell proliferation, migration and colony formation; decreases expression of Keratin18 (KRT18) and increases PTEN and p53 → decreases proteins associated with migration and metastasis in A549 cells	*DR* pro‐oncogenic factors and *UR* anti‐oncogenic factors	KRT18 p53 Metastasis	[94, 162]
	Berberine (isolated compound)	Berberine chloride inhibits colony formations of National Cancer Institute (NCI)‐H460, A549 and NCI‐H1299 NSCLC cells; induces formation of H2AX phosphorylation at Phospho‐Histone H2A.X (Ser139) (known marker for DNA damage) in NCI‐H460 and A549 cells → results in cell apoptosis	*UR* anti‐oncogenic factors	H2AX Apoptosis	[28]
	Berberine (isolated compound)	Berberine increases levels of p53 → inhibits G1 phase of LC tumour cells → initiates apoptosis	*UR* anti‐oncogenic factors	p53 Apoptosis	[190]
*Dimocarpus longan*	Gallic acid (isolated compound)	Gallic acid inhibits EGFR phosphorylation → inhibits PI3K and AKT4 phosphorylation → upregulates wild type p53 in A549 and H292 cells	*DR* pro‐oncogenic factors and *UR* anti‐oncogenic factors	EGFR PI3k p53	[94]
	Gallic acid (isolated compound)	Gallic acid downregulates PD‐L1 → enhances antitumour immunity	*DR* pro‐oncogenic factors	PD‐L1 Apoptosis	[94]
*Fragaria × ananassa*	Fisetin (isolated compound)	Fisetin reduces expression of cyclin‐D, c‐Myc, B Cell lymphoma‐2, COX‐2, MMP‐2, MMP‐9 and CD44; increases expression of CDK inhibitor 1A/B, E‐cadherin (see note) and CDKN2D; increases caspase‐3/9 activity Note: Loss of E‐cadherin with EMT occurs frequently during metastasis and tumour progression	*DR* pro‐oncogenic factors and *UR* anti‐oncogenic factors	Apoptosis EMT Metastasis	[32]
	Fisetin (isolated compound)	Fisetin inhibits tumour endothelial cell migration and angiogenesis in Lewis lung carcinoma; micro‐vessel density significantly reduced when combined with cyclophosphamide	Not investigated	Angiogenesis TME	[97]
	Fisetin (isolated compound)	Combined paclitaxel and fisetin increase A549 cells susceptibility to apoptosis, even though at a low level	Unknown: Cell death possibly due to enlarged mononucleated and multinucleated cells	Unknown pathway	[98]
*Magnoliae officinalis cortex*	Honokiol (isolated compound)	Honokiol → inhibited A549 cell proliferation and migration; enhances A549 cell apoptosis	*DR* pro‐oncogenic factors	Apoptosis PI3K‐AKT	[107]
	Magnolol (isolated compound)	Magnolol → downregulates BCL‐xL expression and upregulates Bad and B‐cell lymphoma extra short (Bcl‐xS) → 100% reduction of viable CH27, H460, H1299 cells (80 μm for 72 h), only 70% reduction in WI‐38 cells	*DR* pro‐oncogenic factors	Bcl‐xL Bad	[106]
*Panax ginseng*	Ginsenoside Rg3 (isolated compound)	Rg3 inhibits cisplatin resistance associated PD‐L1 expression in A549 cells → resumes T cell cytotoxicity; inhibits NF‐κB to promote apoptosis of A549 cells	*DR* pro‐oncogenic factors	NF‐κB Apoptosis	[34]
*Rabdosia rubescens*	Oridonin (isolated compound)	Oridonin → suppresses FAK‐ ERK1/2 signalling pathway → inhibits migration and EMT of small cell LC cells	*DR* pro‐oncogenic factors	FAK‐ERK1/2 EMT	[30]
	Oridonin (nanoparticle encapsulation)	Anisamide‐lipid calcium phosphate nanoparticles loaded with Oridonin → reduces proliferation and viability of A549 cells → reduces tumour volume	Not investigated	Not investigated	[191]
	Oridonin (isolated compound)	Oridonin at concentrations of 28–56 μmol·L^−1^ inhibits human lung cancer cell line SPC‐A1 (SPC‐A1) cell growth; promotes cell apoptosis via downregulation of Bcl‐2 and upregulation of Bax proteins	*DR* pro‐oncogenic factors and *UR* anti‐oncogenic factors	Bcl‐2 Bax	[118]
*Rhei/Rheum palmatum radix et rhizoma*	Emodin (isolated compound)	Emodin reduces viability and induces apoptosis of A549 and H1299 cells via regulating tribbles pseudokinase 3 (TRIB3) expression	*UR* anti‐oncogenic factors	TRIB3	[192]
	Emodin (isolated compound)	Emodin inhibits mTOR and AKT; activates AMPK pathway; increases ROS levels → arrests cell cycles and increases apoptosis in A549 and H460 cell lines	*DR* pro‐oncogenic factors and *UR* anti‐oncogenic factors	AMPK AKT–mTOR	[29]
	Emodin (isolated compound)	Emodin downregulates P‐glycoprotein (Pgp) expression in A549 and H460 cells → reduces cell efflux → enhances cisplatin sensitivity	*DR* pro‐oncogenic factors	P‐glycoprotein	[193]
	Emodin (isolated compound)	Emodin reduces expression of Bcl‐2 and inhibits Group II Secretory Phospholipase A2 (sPLA2‐IIa) and NF‐κB	*DR* pro‐oncogenic factors	sPLA2‐IIa NF‐κB	[29]
	Emodin (isolated compound)	Emodin combined with paclitaxel → increases expression of bax and caspase 3; decreases Bcl‐2, p‐Akt and p‐ERK → enhances apoptosis of A549 cells	*DR* pro‐oncogenic factors and *UR* anti‐oncogenic factors	Apoptosis Bax/caspase 3 Bcl‐2 p‐Akt p‐ERK	[99]
*Taraxacum mongolicum herba*	Whole plant extract	Taraxicum total flavonoids → inhibits proliferation and promotes apoptosis of A549 and H1299 cells; increases CD4, CD8, IL‐2, IL‐3, IFN‐γ, and TNF‐α	*UR* anti‐oncogenic factors	Apoptosis Immune response	[194]
	Taraxasterol (isolated compound)	Taraxasterol downregulates expression of with N‐cadherin, vimentin, snail1, snail2, MMP9; upregulates expression of E‐cadherin → inhibits EMT process → inhibits cell migration of SPC‐A1 and LLC SCLC cell lines Taraxasterol increases Bax, caspase‐9, and poly(ADP‐ribose) polymerase 1 (PARP1) and downregulates Bcl‐2	*DR* pro‐oncogenic factors	Cell migration Apoptosis	[35]
*Vaccinium sect cyanococcus*	Pterostilbene (isolated compound)	Pterostilbene enhances endoplasmic reticulum stress (ERS) signalling Phosphorylated protein kinase R – like endoplasmic reticulum kinase (p‐PERK), Inositol‐Requiring Enzyme 1 Alpha (IRE1), ATF4, CHOP → decreases cell viability and induces apoptosis in A549 & PC9 NSCLC cells	*UR* anti‐oncogenic factors	Apoptosis	[101]
	Pterostilbene (isolated compound)	Pterostilbene decreases CDK and cyclin A and cyclin E levels in H520 cells → induces S phase arrest; stimulates caspase‐3, ‐8 and ‐9 activity → induces apoptosis of H520 cells	*UR* anti‐oncogenic factors	Caspase‐3, 8, 9 Apoptosis	[102]
*Zingiber officinalis rhizoma*	[6]‐Shogaol (Isolated Compound)	[6]‐Shogaol metabolised by A549 cells → activates p53 and PUMA pathways, but not B‐cell leukaemia 3 (Bcl‐3) → cell apoptosis	*UR* anti‐oncogenic factors	p53	[195]

## 4 Phytoceutical loaded nanodelivery systems

### 4.1 The challenge of bioavailability

The bioavailability of compounds is an ever‐present challenge. Molecular properties, hepatic and gut metabolism, intestinal wall absorption, delivery methods, solubility, gastric pH, gut microflora, and excretion all impact the final amount of active ingredient at its target site [36, 140]. Lipinski's ‘Rule of 5’ (Ro5) attempts to pinpoint specific molecular properties that provide maximum bioavailability [36]. These properties include 5 or fewer hydrogen (H) bond donors, 10 or fewer H bond acceptors, a molecular mass of fewer than 500 daltons, a calculated logarithm of the octanol–water partition coefficient log *P*‐value (clogP) of 5 or fewer, and no more than 10 rotatable bonds [36]. However, as Schultz highlights, since the Ro5 concept was first disseminated in 1997, the average molecular weight of oral drugs has increased to over 500 daltons, and there has been a statistically significant increase in the number of H bond acceptors [141]. Additionally, as Benet *et al*. [142] remind us, the Ro5 is only relevant for compounds that are not substrates for active transporters. It is no surprise, then, that as of 2022, 50% of new oral drugs approved by the U.S. Food and Drug Administration do not fit within the Ro5 framework [143]. Without an applicable framework, the bioavailability of novel anticancer phytoceuticals with complex chemical compounds is inadequately defined [140].

Hepatic and gut metabolism primarily involve glucuronidation and cytochrome P450 enzymatic pathways, key mechanisms for removing unwanted substances from the body, ultimately reducing a compound's bioavailability [36, 144, 145]. Glucuronidation can be inhibited by delivering a molecule with a co‐agent such as quercetin, which reduces uridine 5′‐diphospho‐glucuronosyltransferase (UGT) [36, 146]. Curcumin is an example of an antioncogenic phytoceutical with poor solubility and low intestinal absorption [146]. Grill *et al*. [146] identified quercetin, tangeretin, and silibinin as effective co‐agents to improve the bioavailability of curcumin *in vivo* via inhibition of UGT. Piperine has also been shown as an effective adjuvant compound to improve curcumin's bioavailability up to 2000%; however, this function is primarily via an increase in intestinal brush border membrane fluidity, and hence intestinal absorption, rather than UGT inhibition [146, 147]. In addition to metabolic considerations, novel delivery systems that increase bioavailability are also being widely investigated.

Many phytochemicals such as flavonoids, tannins, and terpenoids are highly water soluble, poorly absorbed due to large molecular sizes, and are unable to pass through the lipid membranes of cells [37]. These challenges can be addressed by loading a nanoparticle with the phytochemical compound to deliver the phytochemical to its intended target. [37, 146, 148, 149, 150, 151].

### 4.2 Nanoparticle delivery systems

Nanoparticle delivery systems enable optimised drug delivery, sustained drug release, reduced drug degradation, and decreased toxicity [152]. They include, but are not limited to, solid lipid nanoparticles (SLN), polymeric nanoparticles (PMN), liquid crystalline nanoparticles (LCN), liposomes, dendrimers, and microemulsions [37, 148, 151, 153, 154, 155].

#### 4.2.1 Solid lipid nanoparticles

Solid lipid nanoparticles, first developed in the late 20th century, gained popularity as a delivery system due to their high physiochemical stability and efficient mass production [37]. With a size ranging from 10 to 1000 nm, SLNs contain highly purified triglycerides, which remain mostly solid at room temperature, thereby reducing molecular degradation [37]. SLNs are suitable for a range of delivery routes including oral, parenteral, and transdermal [155]; however, due to the structure of SNLs, they have a poor drug loading capacity and are prone to drug expulsion [150, 156].

#### 4.2.2 Polymeric nanoparticles

Similar in size to SLNs, PMNs are made from natural or artificial biodegradable polymers, although natural materials have the beneficial capacity to deliver multiple components simultaneously and can be targeted to specific sites [37]. The size of PMNs varies greatly, which risks inefficient tissue diffusion and poor surface functionality [157, 158]. Additionally, persistent exposure on living cells may result in a range of adverse effects in humans [159].

#### 4.2.3 Liquid crystalline nanoparticles

Existing in a state between crystalline solid and isotropic liquid, liquid crystals are known as mesophases [37]. Generally classified as either thermotropic (a phase transition due to temperature alone) or lyotropic (a phase transition due to temperature and mesogen concentration), it is the latter form that is most used for delivery systems [160]. Due to their structural resemblance to biological membranes, LCNs can deliver phytoceuticals such as berberine [161, 162, 163, 164, 165, 166, 167], naringenin [168], rutin [169, 170, 171], and zerumbone [172, 173] effectively to promote an interaction between the active molecule and the target site; however, they are costly to produce [37, 174, 175]. Novel low‐cost product methods have been developed by Lee *et al*. [174], who were able to synthesise a pharmaceutical (hepatitis C virus inhibitor) loaded LCN of 100 nm with tissue‐specific (liver) distribution. However, the authors of this study noted challenges. They observed an increase in particle size when stored for more than 1 week, due to the concentration of drug within the particle, leading to acceleration of coalescence and Ostwald ripening. Therefore, it was suggested that the resultant formulations are best used for laboratory investigations within 2–3 days of production [174].

#### 4.2.4 Liposomes

Liposomes are an attractive delivery system, as with one or more phospholipid membranes separated by an aqueous medium, they can simultaneously transport multiple payloads, whether hydrophilic, lipophilic, or both [152]. Recently, Singh *et al*. [176] demonstrated *in vitro* the therapeutic potential of a specialised liposome delivery system loaded with curcumin, which was successful in attenuating expression of proteins associated with cancer cell growth and metastasis. This study utilised PlexoZome®, a liposomal bilayer formulation encompassing curcumin and phosphatidylcholine (CUR‐PLXZ). Results of this study showed that the formulation downregulated the pro‐oncogenic proteins epithelial cell adhesion molecule (EpCAM) and oestrogen receptor alpha (ERα) on A549 cell lines [176].

To deliver an active compound to a specific target, ligands can be attached to the liposome surface [152]. This was demonstrated by Ma *et al*. [177] who assessed *in vitro* the delivery of berberine and magnolol loaded liposomes targeted to the cell surface adhesion receptor CD44 (CD44) expressed on A549 cells. The authors showed that the liposome formulation promoted apoptosis of the A549 cells by upregulating Bax and Caspace‐3 with a concurrent downregulation of Bcl‐2 [178]. Even so, a key challenge of liposome‐based drug delivery is the interaction with biomolecules including low density lipoproteins (LDLs), high density lipoproteins (HDLs), and degradation due to the extracellular protein opsonin [179]. To circumvent these issues, the surface of liposomes can be modified with polyethylene glycol (PEG), otherwise known as PEGylation [180]. PEGylated liposomes have been shown to enhance drug accumulating within tumour tissue, while attenuating side effects [181].

Notwithstanding the challenges that researchers face, advancements in nanoparticle delivery systems are enabling intelligent features such as bespoke particle size, adjusted drug release capacity triggered by specific nanoparticle molecular weight, increased blood circulation time, amplified stability, and even targeting of tumour associated macrophages (TAMs) in order to mitigate tumour proliferation and metastasis [180, 182, 183]. Future production methods will improve and hone precise phytoceutical loading, precision targeting, mitigation of side effects, and enhanced therapeutic outcomes in cancer treatment [182].

## 5 Conclusion and future directions

Lung cancer is the leading cause of cancer deaths worldwide, with NSCLC accounting for the majority of lung cancer cases [1, 3]. The burden of cancer is high, with physical, emotional, and financial stress being key considerations for disease management [3, 15, 16, 20]. Standard treatment protocols such as chemotherapy and radiotherapy have unwanted side effects that reduce the patient's quality of life [184, 185]. Additionally, therapeutic resistance, bioavailability, toxicity, and targeting of cancer drugs is a challenge [21, 36, 37]. To support cancer treatment, a plethora of phytoceutical compounds has been identified to target specific cancer‐related pathways known as the hallmarks of cancer. As shown in Table 1, phytoceuticals may be beneficial to cancer therapy by inhibiting pro‐oncogenic pathways, ameliorating antioncogenic pathways, or by enhancing the mechanisms of pharmaceutical drugs. However, many phytoceuticals have poor bioavailability. Various nanoparticle delivery systems have been shown to improve the bioavailability of compounds and novel production methods are enabling multiple compounds to be delivered simultaneously and with tumour specificity [182].

Cancer is a multifaceted disease [186], and the development of phytoceutical nanoparticle delivery systems is just as complex. However, as shown in this review, there is potential for new production methods of nanoparticle systems loaded with phytoceuticals such as artemisinin, berberine, emodin, fisetin, and oridonin to become a beneficial adjunct therapy in mitigating NSCLC.

## Conflict of interest

AHM is employed as a Research and Documentation Consultant with Panaxea International, an herbal medicine company producing phytoceutical products including some to support complimentary cancer care. Panaxea International was not involved in the production, nor benefits from the publication of this article.

## Author contributions

All authors contributed to the article conception and design. Article structure was performed by AHM, KRP, GDR, and KD. The body of the article written by AHM. Editing and revisions of the article were completed by AHM, KRP, SK, TES, SY, DKC, JA, and KD. Artwork for figures was contributed by AHM, GDR, and DKC. All authors read and approved the final article.

### Peer review

The peer review history for this article is available at https://www.webofscience.com/api/gateway/wos/peer‐review/10.1002/1878‐0261.13764.
